# Susceptibility Weighted Imaging: A New Tool in the Diagnosis of Prostate Cancer and Detection of Prostatic Calcification

**DOI:** 10.1371/journal.pone.0053237

**Published:** 2013-01-07

**Authors:** Yan Bai, Mei-Yun Wang, Yan-Hong Han, She-Wei Dou, Qing Lin, Ying Guo, Wei Li, De-Gang Ding, Jian-Ping Dai, Wei Qin, Da-Peng Shi, Jie Tian, Yong-Ming Dai

**Affiliations:** 1 Department of Radiology, Henan Provincial People's Hospital, Zhengzhou, Henan, China; 2 Department of Urinary Surgery, Henan Provincial People's Hospital, Zhengzhou, Henan, China; 3 MRI, Siemens Healthcare, Shanghai, China; 4 Institute of Automation, Chinese Academy of Sciences, Beijing, China; 5 Life Science Research Center, School of Sciences and Technology, Xidian University, Xi’an, Shanxi, China; University of California San Francisco, United States of America

## Abstract

**Background:**

Susceptibility weighted imaging (SWI) is a new MRI technique which has been proved very useful in the diagnosis of brain diseases, but few study was performed on its value in prostatic diseases. The aim of the present study was to investigate the value of SWI in distinguishing prostate cancer from benign prostatic hyperplasia and detecting prostatic calcification.

**Methodology/Principal Findings:**

23 patients with prostate cancer and 53 patients with benign prostatic hyperplasia proved by prostate biopsy were scanned on a 3.0T MR and a 16-row CT scanner. High-resolution SWI, conventional MRI and CT were performed on all patients. The MRI and CT findings, especially SWI, were analyzed and compared. The analyses revealed that 19 out of 23 patients with prostate cancer presented hemorrhage within tumor area on SWI. However, in 53 patients with benign prostatic hyperplasia, hemorrhage was detected only in 1 patient in prostate by SWI. When comparing SWI, conventional MRI and CT in detecting prostate cancer hemorrhage, out of the 19 patients with prostate cancer who had prostatic hemorrhage detected by SWI, the prostatic hemorrhage was detected in only 7 patients by using conventional MRI, and none was detected by CT. In addition, CT demonstrated calcifications in 22 patients which were all detected by SWI whereas only 3 were detected by conventional MRI. Compared to CT, SWI showed 100% in the diagnostic sensitivity, specificity, accuracy, positive predictive value(PPV) and negative predictive value(NPV) in detecting calcifications in prostate but conventional MRI demonstrated 13.6% in sensitivity, 100% in specificity, 75% in accuracy, 100% in PPV and 74% in NPV.

**Conclusions:**

More apparent prostate hemorrhages were detected on SWI than on conventional MRI or CT. SWI may provide valuable information for the differential diagnosis between prostate cancer and prostatic hyperplasia. Filtered phase images can identify prostatic calcifications as well as CT.

## Introduction

Prostate cancer is the fifth most common cancer [1] and become a major worldwide public health problem [2], which causes 6% of cancer deaths in men [1]. MRI has been a useful tool to detect PCa, but it’s still difficult to distinguish Prostate cancer from benign prostatic hyperplasia sometimes, especially when the tumor is located in the central zone of prostate. In addition, prostatic calcification is also difficult to be detected clearly by conventional magnetic resonance imaging (MRI) as the signal intensity (SI) of calcification is varied [3], [4] and the size of calcification is usually very small.

Susceptibility weighted imaging (SWI) is a new MRI technology which reflects the magnetic susceptibility of tissue and is exquisitely sensitive to paramagnetic deoxygenated blood products such as deoxyhemoglobin, methemoglobin and haemosiderin [5]. It includes not only magnitude information but also useful phase information, which was usually ignored in most diagnostic MR imaging. To make good use of phase information, though phase and magnitude images separately are also critical pieces of information, Dr. Haacke et al. combined the filtered phase and the magnitude information and thus created a new susceptibility-weighted magnitude image, i.e. SWI [6]. SWI was proved to be much more sensitive in detecting microbleeds in brain thangradient-recalled echo (GRE)- and GRE-type single-shot echoplanar imaging (GREI, GRE-EPI) [7]. Because diamagnetic calcification and paramagnetic blood products present opposite signal features on the filtered phase images, it is easy to distinguish calcification from hemorrhage by using filtered phase image [8]. Thus SWI and filtered phase image has been widely used in the detection of intracerebral microbleed and display of calcification in central nervous system [9]–[12]. However, there are no reports on SWI in prostate so far. This study investigated the value of high-resolution SWI and filtered phase image in distinguishing prostate cancer from benign prostatic hyperplasia and detecting calcification by comparing with conventional MR and CT images.

## Materials and Methods

### Ethics Statement

This study was approved by the hospital review boards of Henan Provincial People’s Hospital. Written informed consent was obtained from all patients. All research procedures were conducted in accordance with the Declaration of Helsinki.

### Study Population

This was a prospective study enrolling 76 patients with prostate diseases in Henan Provincial People's Hospital from June 2011 to September 2012. Transrectal ultrasonography (TRUS)-guided prostate biopsy proved 23 patients with prostate cancer (age range 55–91 years, average age 71 years) (Table 1) and 53 patients with benign prostatic hyperplasia (age range 49–84 years, average age 68 years). High-resolution SWI, conventional MRI and CT were performed on all patients prior to prostate biopsy, transurethral resection, endocrine therapy, brachytherapy, radiotherapy or drug treatment for the prostate disease.

**Table 1:** Characteristics of 23 male patients with prostate cancer.

Case No./age (year)	SWI	Location of Pca
1/79	Hemorrhage	Central Zone
2/78	Hemorrhage	Peripheral Zone
3/68	Hemorrhage	Central Zone
4/91	Hemorrhage	Peripheral Zone
5/78	Hemorrhage	Peripheral Zone
6/64	Hemorrhage	Peripheral Zone
7/55	Hemorrhage	Central Zone
8/72	Hemorrhage	Peripheral Zone
9/76	Hemorrhage	Peripheral Zone
10/79	Hemorrhage	Peripheral Zone
11/70	Hemorrhage	Peripheral Zone
12/71	Hemorrhage	Peripheral Zone
13/70	Hemorrhage	Peripheral Zone
14/56	Hemorrhage	Peripheral Zone
15/68	Hemorrhage	Peripheral Zone
16/73	Negative	Peripheral Zone
17/76	Negative	Peripheral Zone
18/71	Negative	Peripheral Zone
19/66	Hemorrhage	Peripheral Zone
20/72	Hemorrhage	Peripheral Zone
21/60	Hemorrhage	Peripheral Zone
22/71	Negative	Central Zone
23/69	Hemorrhage	Peripheral Zone

#### Imaging acquisition

MRI was performed on a Siemens 3T scanner (Magnetom Trio, Siemens Medical Solutions, Erlangen, Germany) with a pelvic array phased coil (Siemens Medical System).

SWI is a three-dimensional fast low-angle gradient-echo (GRE) sequence. The imaging parameters of SWI for prostate are as follows: field of view (FOV) 300×300 mm^2^, matrix 282×512, TR (repetition time)/TE (echo time) = 22/12 milliseconds (ms), 20° flip angle, and 3 mm slice thickness. The acquisition time was 3 minutes and 36 seconds. The SWI images were created by using the magnitude and phase images [13]. The phase image was high pass filtered (by using a 64×64 exclusion of low-spatial-frequency information) to remove much of the spine’s low spatial frequency background static field variation. A phase mask was created by setting all positive phase values (between 0° and 180°) to unity and normalizing the negative-phase values ranging from 0° to −180° to a gray scale of values ranging linearly from 1 to 0, respectively. This normalized phase mask was multiplied four times against the original magnitude image and yielded images that maximized the negative signal intensities of the regions containing deoxygenated blood and increased the contrast between regions containing deoxygenated blood and the surrounding tissue. Finally, a minimum intensity projection over two sections was performed to display the processed data by using contiguous 4-mm-thick sections in the transverse plane.

Conventional MRI was performed with a fast spin-echo (FSE) sequence. The imaging parameters were as follows:

Axial T1- weighted image (WI): field of view (FOV) 300×300 mm^2^, matrix 288×320, TR (repetition time)/TE (echo time) = 700/11 milliseconds (ms), 150° flip angle, and 3 mm slice thickness. The acquisition time was 3 minutes and 25 seconds.

Axial T2WI: FOV 300×300 mm^2^, matrix 272×320, TR/TE = 4000/87 ms, 140° flip angle, and 3 mm slice thickness. The acquisition time was 3 minutes and 54 seconds.

Sagittal T2WI: FOV 250×250 mm^2^, matrix 272×320, TR/TE = 4000/87 ms, 140° flip angle, and 3 mm slice thickness. The acquisition time was 3 minutes and 54 seconds.

Coronal T2WI: FOV 250×250 mm^2^, matrix 192×256, TR/TE = 4000/104 ms, 145° flip angle, and 4 mm slice thickness. The acquisition time was 2 minutes and 26 seconds.

CT was performed on a 16-row CT scanner (Brilliance 16, Philips Medical Systems). The imaging parameters are as follows: 120KV tube voltage, 250 mA tube current, and 3 mm thickness.

#### Histopathologic examination

Each patient underwent transrectal ultrasound-guided sextant biopsies after completion of the MRI and CT examination within 10 days. The pathological results revealed that 23 patients had prostate cancer and 53 patients had benign prostatic hyperplasia.

#### Imaging analysis

Two radiologists with 11 and 15 years’ diagnostic experience, respectively, blinded to the histopathologic results analyzed all images. Tumorous and non-neoplastic areas were determined on the MR images in patients with prostate cancer. They observed the hemorrhagic foci and calcification in the prostate and discussed the final results when disaccordance appeared.

#### Statistical analysis

SPSS 17.0 statistical software was used to analyze data. Fisher's exact test was used to analyze the hemorrhagic manifestations on SWI between prostate cancer and benign prostatic hyperplasia group. A p value of less than 0.05 was considered significant. The sensitivity, specificity, accuracy, negative predictive values (NPV) and positive predictive values (PPV) at SWI and conventional MRI in detecting calcifications in prostate were evaluated using CT as the gold standard.

## Results

**Figure 1:**
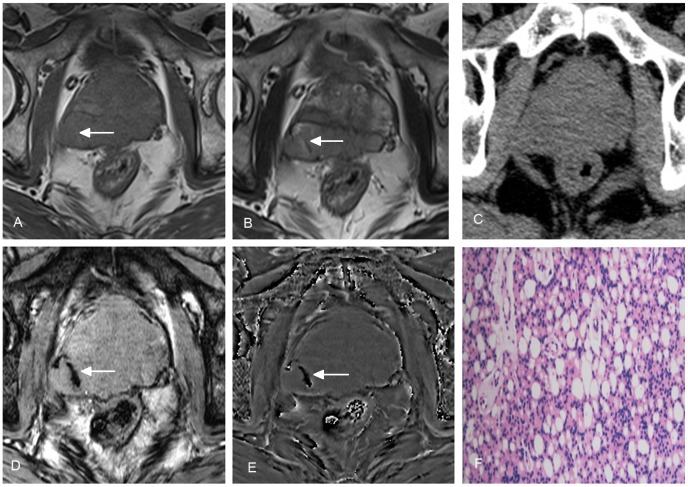
A 64-year-old man with prostate cancer in peripheral zone of the prostate. Heterogeneous signal on conventional T1WI (A) and T2WI (B) (arrows) indicates tumor hemorrhage. No hemorrhage is demonstrated on CT (C). The tumor hemorrhage was also seen with SWI (D) and filtered phase image (E) (arrows). Histopathologic examination confirmed the diagnosis of prostate cancer (F).

**Figure 2:**
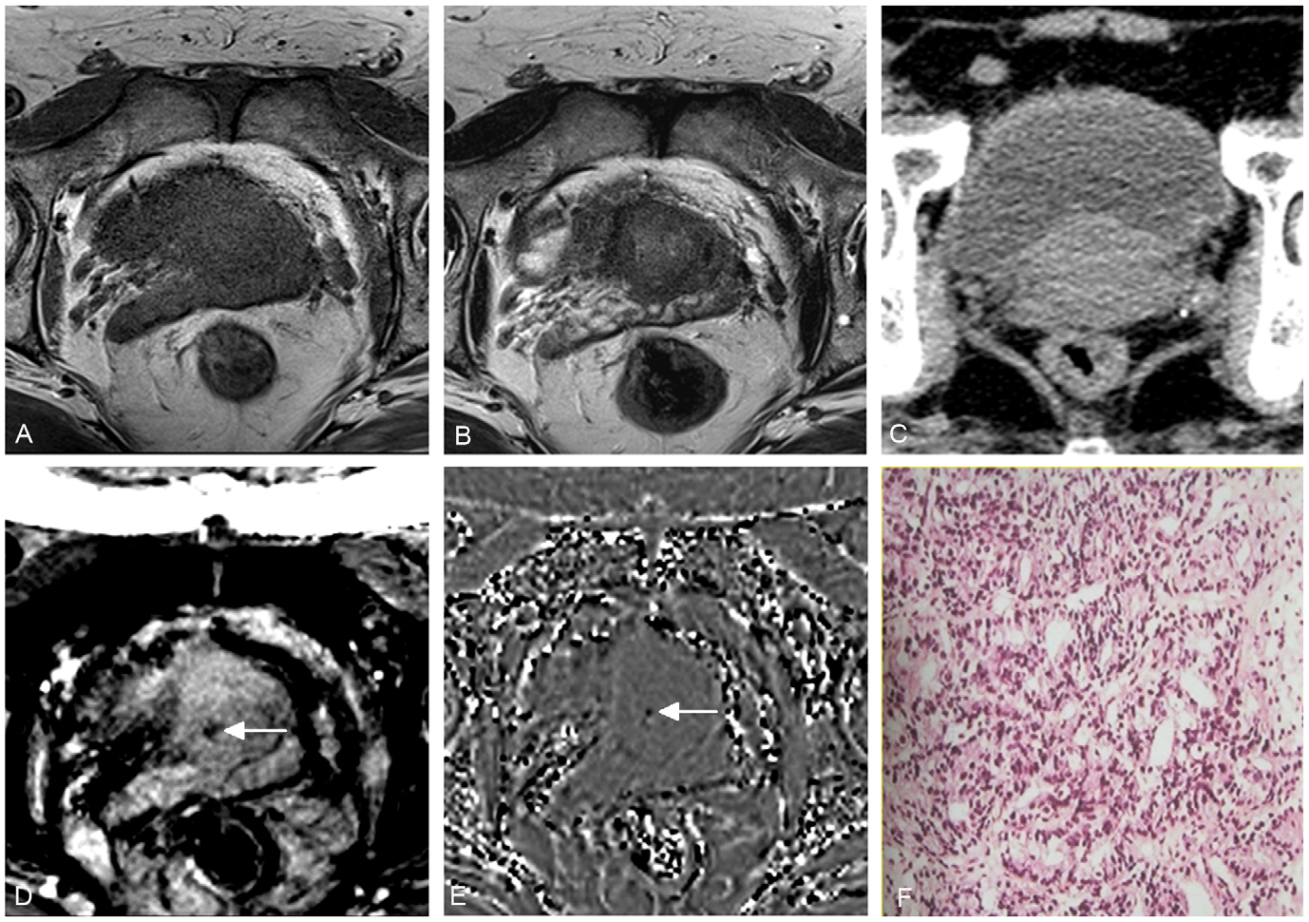
A 55-year-old man with prostate cancer in central zone of the prostate. No tumor hemorrhage is demonstrated on conventional T1WI (A), T2WI (B) and CT (C), but low signal within tumor on SWI (D) and filtered phase image (E) (arrows) indicates tumor hemorrhage. Histopathologic examination confirmed the diagnosis of prostate cancer (F).

**Figure 3:**
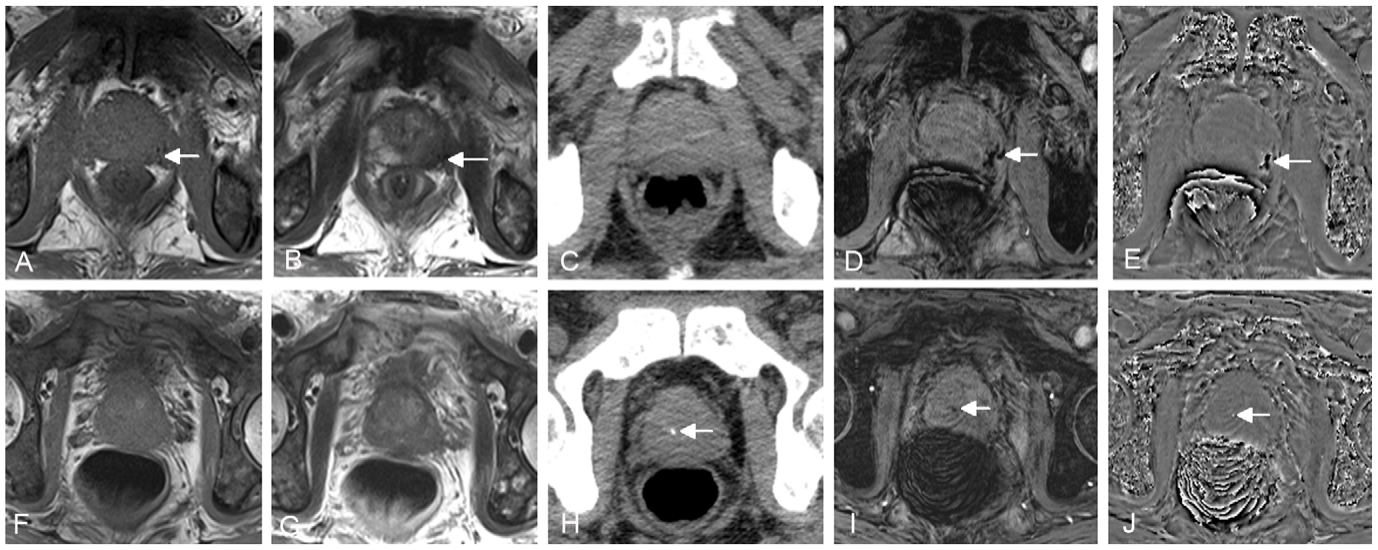
A 66-year-old man with prostate cancer in peripheral zone of the prostate. Low signal on conventional T1WI (A) and T2WI (B) (arrows) indicates tumor hemorrhage. No hemorrhage is demonstrated on CT (C). The tumor hemorrhage was also seen with SWI (D) and filtered phase image (E) (arrows). The images in second row come from another slice of the same patient. No prostatic calcification is demonstrated on conventional T1WI (F) and T2WI (G), but dot-like high density on CT (H), low signal on SWI (I) and high signal on filtered phase image (J) (arrows) indicates calcificaiton.

The tumor lesions of 19 patients with prostate cancer were located in the peripheral zone of the prostate, only 4 cases were within the central region. In 19 out of 23 patients (82.6%) with prostate cancer, hemorrhage was detected within the tumorous areas (16 patients with prostate cancer in the peripheral zone and 3 patients with tumor lesions in the central zone) by SWI (Table 1). However, small hemorrhage was detected only in 1 patient out of 53 (1.9%) patients with benign prostatic hyperplasia. Fisher's exact test showed significant difference between prostate cancer and benign prostatic hyperplasia in the detection of hemorrhage within lesions (P<0.05). Out of the 19 patients with prostate cancer who had prostatic hemorrhage detected by SWI, only 7 patients had prostatic hemorrhage on conventional MRI (Figs. 1, 2, 3). Bleeding was not detected in all the patients by using CT. More importantly, the tumor lesions of 4 patients with prostate cancer were located in the central zone of the prostate in this study, and tumor hemorrhage were detected in 3 patients by SWI.

**Figure 4:**
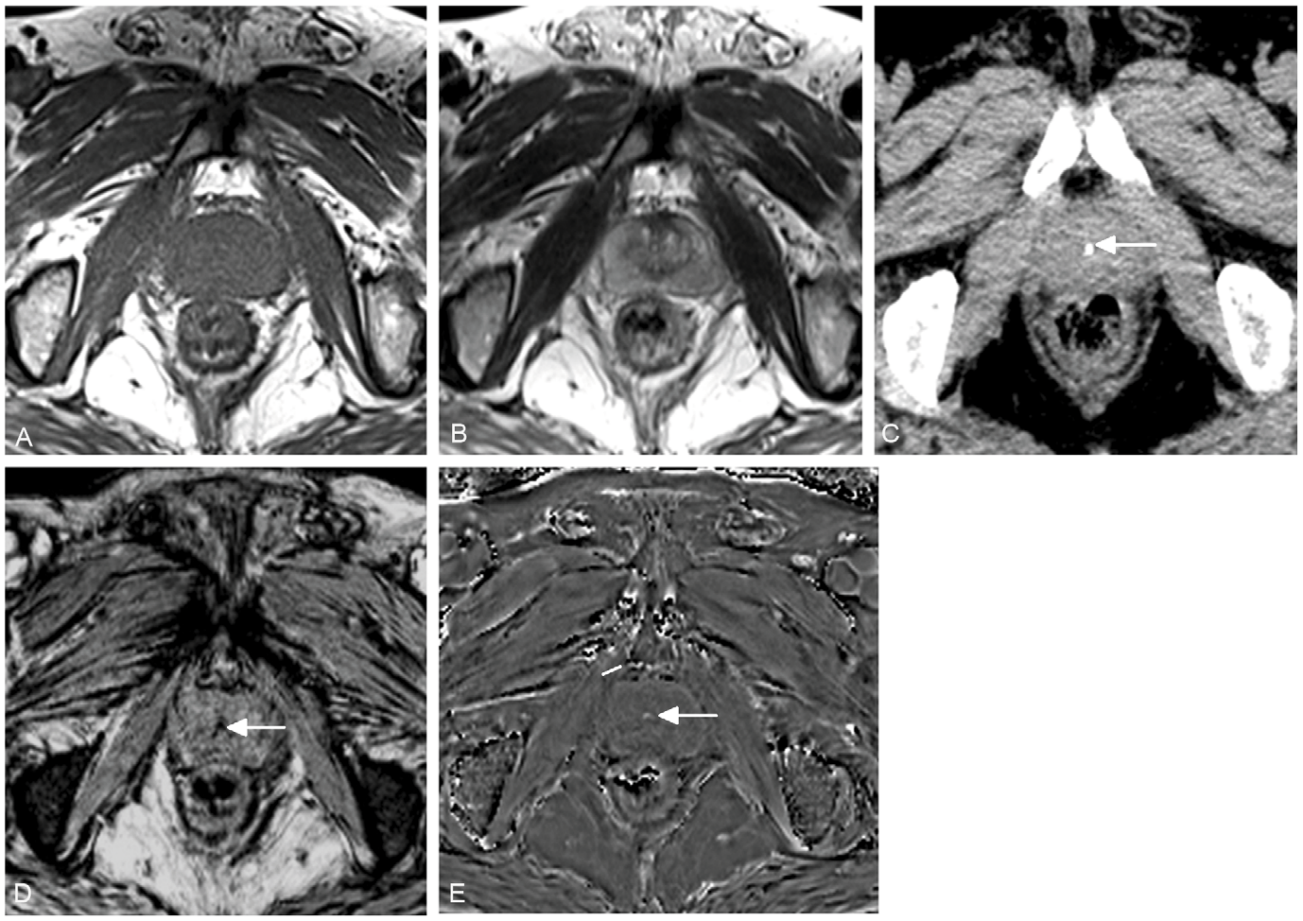
A 62-year-old man with benign prostatic hyperplasia. No prostatic calcification is demonstrated on conventional T1WI (A) and T2WI (B), but dot-like high density on CT (C), low signal on SWI (D) and high signal on filtered phase image (E) (arrows) indicates calcificaiton.

The calcificatinos were detected in 22 patients by CT, including 5 out of 23 patients with prostate cancer and 17 out of 53 patients with benign prostatic hyperplasia.When MRIs were used, the calcifications were detected in all the 22 patients by SWI whereas in only 3 by routine MRI (Fig. 3, 4). Compared to CT, SWI showed 100% in the diagnostic sensitivity, specificity, accuracy, positive predictive value and negative predictive value in detecting calcifications in prostate but conventional MRI demonstrated 13.6% in diagnostic sensitivity, 100% in specificity, 75% in accuracy, 100% in positive predictive value and 74% in negative predictive value.

## Discussion

SWI is a new MRI technique which is more sensitive than CT, conventional MR and T2*WI GRE sequences in detecting paramagnetic blood products such as deoxyhemoglobin, methemoglobin and haemosiderin in central nervous system [5]. It has been widely used in detecting microbleeds in a variety of brain diseases such as brain trauma, stroke and vascular malformation [8]–[11]. In addition, SWI in spinal cord trauma has also been investigated by our team and was proved valuable in detecting spinal cord hemorrhage [14]. Some recent studies in glioma have explored SWI’s value and found that it’s helpful in tumor grading and patient management strategies [15], [16]. But so far no studies have been done on the value of SWI in prostate cancer and other prostate diseases.

As an advanced imaging technique, MRI has been gaining acceptance as an important tool in the evaluation of prostate diseases. T2WI is an important traditional sequence for the diagnosis of prostate cancer in the prostate peripheral zone but not specific. It is easy to distinguish the cancerous area which presents hypointense on T2WI from the uniform hyperintense background in the prostate peripheral zone. However, other changes such as prostatitis and fibrosis also can appear hypointense on T2WI [17]. Furthermore, although most of cancers arise in the peripheral zone of the prostate, up to 30% of prostate cancers occur within the central region [18]. It is more difficult to discriminate malignant from benign prostatic hyperplasia because transition zone cancer is the site of origin of benign prostatic hyperplasia, which can have a heterogeneous appearance [19]. Although diffusion weighted imaging (DWI), magnetic resonance spectroscopy (MRS) and dynamic contrast enhanced (DCE) imaging may provide supplementary information for the diagnosis of prostate disease, as each method has its own deficiencies, overlap appears in the differential diagnosis between prostatic cancer and benign prostatic hyperplasia [20].

This study investigated the potential of SWI in distinguishing prostate cancer from benign prostatic hyperplasia. In 23 patients with prostate cancer, hemorrhage was detected in 19 patients within the tumorous areas by SWI (82.6%). However, small hemorrhage was detected only in 1 patient out of 53 (1.9%) patients with benign prostatic hyperplasia by SWI. The hemorrhage ratio within the lesions had significant difference between the two diseases. From this result, it seemed that prostate cancer may be more prone to bleeding than benign prostatic hyperplasia. The possible reason may be that the prostate cancer tissue has higher microvessel density (MVD) which was caused by increased vascular endothelial growth factor (VEGF) expression than normal prostate or benign prostatic hyperplasia tissue [21], [22]. Over-expression of Id-1 (inhibitor of differentiation/DNA synthesis) which belongs to the Id family of helix–loop–helix proteins is a key factor in promoting angiogenesis through activation of the VEGF in prostate cancer cells [23]. The new microvessels in tumors generally differ from those of noncancerous tissue. Although tumorous area contains a greater number of vessels than non-neoplastic area, the vessel surface area density is reduced. This reflected that the size and shape of tumor microvessels tend to be broad and less branched than those in normal tissue [24]. It may lead to bleeding in prostate cancer as the new microvessels are more fragile and irregular with increased permeability and higher proliferation rate than that of normal endothelial cells [21]. Blood ﬂow measured in tumor-containing prostate is generally higher than that in prostates tissue containing benign prostatic hyperplasia [25]. The differences between prostate cancer and benign prostatic hyperplasia in microvascular structure and hemodynamics may be the main reasons of high incidence of prostate cancer bleeding. In addition, out of the 19 patients with prostate cancer who had prostatic hemorrhage detected by SWI in this study, conventional MRI only detected prostatic hemorrhage in 7 patients. It suggested that SWI is more sensitive in detecting prostatic hemorrhage than conventional MRI. More importantly, the tumor lesions of three patients with prostate cancer were located in the central zone of the prostate in this study, and tumor hemorrhage were all detected in these three patients by SWI. This finding would be very helpful for the accurate diagnosis of prostate cancer in central zone. Although not all patients with prostate cancer demonstrated hemorrhage on SWI, the supplementary information provided by SWI may be valuable for the diagnosis of prostate cancer. As the sample size of this study was small, more larger studies need to be performed to further prove these results.

Prostatic calcification is frequently encountered in urological practice. Some reports revealed that small, multiple calcifications are a normal, often incidental ultrasonographic finding in the prostate and represent a result of age rather than a pathologic entity. However, larger prostatic calcification may be related to underlying inflammation and require further evaluation and possible treatment [26], [27]. Traditionally, CT is thought the gold standard for detection of calcification which can be determined with Hounsfield units (Hu) above 100 [28]. On routine MRI, the signal of calcification is varied because of diverse calcium compounds and difficult to distinguish it from hemorrhage. Therefore, the ability of CT in detecting calcification is far greater than conventional MRI. With the development of MRI techniques, filtered phase image has become a very sensitive technique in detecting calcification in brain [8], but no study was performed to investigate its value in detecting prostatic calcification. This study demonstrated that filtered phase image has equal efficiency in detecting prostatic calcification as CT and far higher efficiency than routine MRI. The mechanism may be that filtered phase image is exquisitely sensitive to differences in local magnetic susceptibility, which can be induced by both hemorrhage and calcification [5]. Both calcification and hemorrhage show low signal on SWI, but present opposite signal features on filtered phase images. Usually calcification is high signal or mixed signal dominated by high signal but hemorrhage displays as low signal or mixed signal dominated by low signal on filtered phase images [29]. So filtered phase image is useful in distinguishing calcification from hemorrhage. To overcome ill-posed nature of the inverse filter and improve susceptibility quantification, Dr. Haacke et al. introduced a form of susceptibility mapping to produce an image of veins from phase data [30]. Both simulations and human studies have demonstrated that this approach can dramatically reduce streaking artifacts and improve the accuracy of susceptibility quantification inside the structures of interest such as veins or other brain tissues [31]. In the future, it may be possible to use this approach to evaluate quantitatively microbleeds and calcifications and allow a straightforward identification of calcification.

The major limitation of this study is that the histopathologic examination were all performed by biopsy instead of prostate resection. So the tumor hemorrhage on SWI was not directly proved by histopathologic examinations. In addition, the sample size in this study is not very large so we did not evaluate the incidence of tumor bleeding at different stages in patients with prostate cancer. Future studies may need to get more reliable results and investigate the potential of SWI in the prostate cancer staging.

In conclusion, our results indicate that SWI is more sensitive in the detection of prostate microbleeding and may be helpful in the differential diagnosis between prostatic cancer and benign prostatic hyperplasia. Filtered phase images can identify prostate calcifications as well as CT. More studies with larger sample size are needed to get more reliable results for clinical practice in the future.
